# Focus on the Heart: Alcohol Consumption, HIV Infection, and Cardiovascular Disease

**Published:** 2010

**Authors:** Matthew S. Freiberg, Kevin L. Kraemer

**Keywords:** Alcohol consumption, alcohol use disorder, heavy drinking, alcohol and other drug effects and consequences, human immunodeficiency virus, antiretroviral therapy, combination antiretroviral therapy, cardiovascular disease, coronary heart disease, stroke

## Abstract

With the advent of effective antiretroviral therapy, people infected with HIV have a longer life expectancy and, consequently, are likely to develop other chronic conditions also found in noninfected people, including cardiovascular disease (CVD). Alcohol consumption, which is common among HIV-infected people, may influence the risk of CVD. In noninfected adults, moderate alcohol consumption can reduce the risk of coronary heart disease (CHD), heart attacks, and the most common type of stroke, whereas heavy drinking increases the risk of these cardiovascular events. These relationships can be partially explained by alcohol’s effects on various risk factors for CVD, including cholesterol and other lipid levels, diabetes, or blood pressure. In HIV-infected people, both the infection itself and its treatment using combination antiretroviral therapy may contribute to an increased risk of CVD by altering blood lipid levels, inducing inflammation, and impacting blood-clotting processes, all of which can enhance CVD risk. Coinfection with the hepatitis C virus also may exacerbate CVD risk. Excessive alcohol use can further enhance CVD risk in HIV-infected people through either of the mechanisms described above. In addition, excessive alcohol use (as well as HIV infection) promote microbial translocation—the leaking of bacteria or bacterial products from the intestine into the blood stream, where they can induce inflammatory and immune reactions that damage the cardiovascular system.

Currently, more than 60 different medical conditions and 4 percent of the global health burden of disease are caused at least in part by, or are attributable to, alcohol consumption (Room et al. 2005). In the United States, 62.5 percent of adults consume alcohol and 17.6 million have an alcohol use disorder (AUD) (Centers for Disease Control and Prevention 2003; Grant et al. 2004). Because of this widespread and, in many cases, excessive alcohol use, alcohol consumption is associated with the two leading causes of death in the United States—cardiovascular disease (CVD)^1^ and cancer (Mokdad et al. 2005).

Alcohol consumption and AUDs also are common among adults infected with the human immunodeficiency virus (HIV) (Conigliaro et al. 2003; Cook et al. 2001). With the advent of antiretroviral therapy and, as a result, an increasing life expectancy in this population (Palella et al. 1998), chronic diseases such as coronary heart disease (CHD) have become a prevalent and important health issue facing adults with HIV(Friis-Moller et al. 2003*b*; Holmberg et al. 2002, 2004; Klein et al. 2002). For example, among HIV-infected participants in the Veterans Aging Cohort Study (VACS), hazardous drinking and AUDs were independently associated with an increased prevalence of CVD, even after adjusting for traditional CVD risk factors, such as cholesterol levels or coexisting diabetes (Freiberg et al. 2010). Furthermore, among HIV-infected people with an AUD, those who were coinfected with the hepatitis C virus (HCV) had an even higher prevalence of CVD (Freiberg et al. 2007). However, the mechanism(s) by which alcohol use and HCV infection may influence cardiovascular risk and other chronic diseases among HIV-infected people remain unknown.

This article explores the relationships between alcohol use, HIV infection, and CVD. After reviewing the association between alcohol use and CVD among HIV-uninfected adults and the relationship between HIV infection and CVD, the article examines the role of alcohol consumption in CVD among HIV-infected adults. It concludes with a discussion of possible mechanisms underlying alcohol’s association with CVD among HIV-infected adults.

## Alcohol Use and CVD among HIV-Uninfected Adults

CVD refers to any type of disease involving the heart or blood vessels. It comprises a variety of conditions, including CHD (which involves the blood vessels supplying the heart muscle), stroke (which involves blood vessels in the brain), congestive heart failure (which refers to any problem with the structure or function of the heart that interferes with adequate blood supply to the body), and others. Alcohol consumption can either positively or negatively influence the risk of any of these conditions.

### Alcohol Consumption and CHD

In patients with CHD, the blood vessels of the heart muscle cannot supply the heart and surrounding tissues with enough blood—for example, because they are blocked by build-up of fatty materials (e.g., cholesterol) on the walls of the blood vessels. As a result, the heart muscle cannot function properly and thus cannot pump sufficient blood to the rest of the body.

For people without HIV who consume alcohol, CHD risk is strongly associated with the quantity and pattern of alcohol consumption. This association typically is described as a J-shaped curve (Corrao et al. 2000):

- For abstainers, the relative risk (RR) of CHD is set at 1.0, which is an intermediate level.
- Moderate drinkers, who consume 0 to 20 g alcohol, or less than two standard drinks^2^ per day, have the lowest risk, with an RR = 0.8 (95% confidence interval [CI] = 0.78–0.83).
- Heavy drinkers who consume more than 89 g alcohol, or more than six standard drinks per day have the highest risk of CHD, with an RR = 1.05 (95% CI = 1.00–1.11).

The same general relationship has been found with respect to heart attacks (i.e., myocardial infarctions [MIs]). Thus, drinkers who consume one to three drinks (10.0 to 29.9 grams of alcohol) on 3 to 4 days or 5 to 7 days per week have fewer MIs (RR = 0.68, 95% CI = 0.55–0.84 and RR = 0.63, 95% CI = 0.54–0.74, respectively) than people who consume alcohol less than once per week (Mukamal et al. 2003). In contrast, men who drink nine drinks or more per day have a significantly elevated risk for a major coronary event (RR = 2.40; 95% CI = 1.17–4.93) compared with those who do not drink (McElduff and Dobson 1997).

### Alcohol Consumption and Stroke

The relationship between alcohol and stroke is difficult to assess. There are two general categories of stroke:

- Ischemic strokes, which account for about 87 percent of all cases, are caused by blockage of a blood vessel in the brain, resulting in decreased blood supply to the brain regions reached by that vessel.
- Hemorrhagic strokes, which account for about 13 percent of cases, are caused by rupture of a blood vessel in the brain, flooding the surrounding brain tissue with blood and disrupting normal blood supply to certain brain regions.

Multiple factors can influence the risk for either type of stroke, including high blood pressure (i.e., hypertension) and an abnormal heart rhythm involving the two upper chambers of the heart (i.e., atrial fibrillation) (Klatsky 2005).

As with CHD, a J- or U-shaped association curve exists between alcohol and ischemic stroke (Sacco et al. 1999). Thus, compared with no alcohol consumption, consumption of two drinks per day was associated with a lower odds ratio (OR) for ischemic stroke (OR = 0.51, 95% CI = 0.39–0.67), whereas consumption of seven or more drinks per day was associated with a higher OR (OR = 2.96, 95% CI = 1.05–8.29). To date, no clear mechanism has been identified that explains this association. However, several factors may play a role, including the improved lipid profiles, reduced insulin resistance, and favorable blood clotting factor profiles associated with moderate alcohol consumption. Even genetic factors, such as the presence of a gene variant called *apolipoprotein E4*^3^ (*APOE4*), may influence the association between moderate alcohol consumption and reduced risk of stroke (Mukamal et al. 2005).

Heavier alcohol consumption (more than 60 grams per day) also is associated with an increased risk of hemorrhagic stroke (RR = 2.18, 95% CI = 1.48–3.20) (Reynolds et al. 2003). This association may be mediated by an alcohol-related increase in blood pressure, which is an important risk factor for hemorrhagic stroke (Klatsky 2005). Moreover, heavy drinking may interfere with normal blood-clotting processes (i.e., have an antithrombotic effect), which also may also exacerbate the risk of bleeding in the brain (i.e., intracranial bleeding).

### Alcohol Consumption and Congestive Heart Failure

The association between moderate alcohol consumption and congestive heart failure has not been investigated thoroughly; thus, data are sparse and the results inconsistent (Djousse and Gaziano 2008). For example, one study (Djousse et al. 2009) suggests that moderate alcohol consumption in addition to healthy lifestyle behaviors is associated with a lower lifetime risk of heart failure. Heavy alcohol consumption, however, often is associated with deterioration of heart muscle function (i.e., cardiomyopathy), which can lead to heart failure (Lazarevic et al. 2000).^4^

### Alcohol and Traditional Risk Factors for CHD

The relationships between alcohol consumption and various CHD risk factors (e.g., cholesterol or triglyceride levels, diabetes, and blood pressure) among people without HIV have been well characterized:

- The levels of “good” cholesterol (i.e., high-density lipoprotein [HDL] cholesterol) increase in a dose-dependent manner with increasing alcohol consumption (Gaziano et al. 1993).
- The levels of other, harmful fat-like molecules (i.e., triglycerides) in the blood are higher in heavy drinkers (Rimm et al. 1999) but can be lower in moderate drinkers compared with abstainers (Mayer et al. 1993).
- Moderate alcohol consumption (i.e., 30.0 to 49.9 g per day, or two to three standard drinks) is associated with a reduced incidence of diabetes (RR = 0.61, 95% CI = 0.44–0.91) (Rimm et al. 1995) and increased insulin sensitivity (Davies et al. 2002).
- With respect to blood pressure, most studies suggest that all levels of alcohol consumption, but particularly heavy consumption, result in either no change or increased blood pressure (Klatsky 1995). One study (Gillman et al. 1995), however, found a beneficial effect of light to moderate alcohol consumption (i.e., one to less than two drinks per day) on blood pressure.
- Alcohol consumption also can influence the levels of various proteins that can indicate the presence of inflammation and which can be associated with an increased risk of CHD. Moderate alcohol consumption is associated with lower levels of an inflammatory marker called C-reactive protein (Albert et al. 2003) as well as of other inflammatory markers (Sierksma et al. 2002). Conversely, heavier consumption is associated with higher levels of C-reactive protein (Imhof et al. 2001).

All of these findings indicate pathways through which moderate alcohol consumption may contribute to a reduced risk of CVD as well as support the observation that heavy alcohol consumption is associated with an increased risk of CVD.

## HIV Infection and the Risk of CVD

### Influence of HIV Viral Load on Risk

The severity of a viral infection can be reported using a measure called the viral load, which reflects the number of virus particles in a given volume of blood (e.g., per milliliter), with higher viral loads indicating more severe infection. By tracking a patient’s viral load over time, clinicians can monitor disease progression or the effectiveness of treatment.

The most current evidence suggests that higher HIV viral loads are associated with increased cardiovascular risk. For example, researchers in the Strategies for Management of Antiretroviral Therapy Trial (SMART) studied the risk of CVD in HIV-infected patients receiving different types of combination antiretroviral therapy (CART)^5^ resulting in different viral loads (El-Sadr et al. 2006). The analysis found that continuous CART, which resulted in a suppression of viral load, was associated with a lower CVD risk (hazard ratio [HR] = 1.6, 95% CI = 1.0–2.5) than episodic CART, which aimed to maintain the virus’ target cells—a type of immune cell called CD4 cell—at a specific level, even if viral load was higher.

Since the introduction of CART, study findings have suggested that HIV infection also is associated with increased risk for, and faster progression of, a very early stage of CVD known as subclinical atherosclerosis (Hsue et al. 2004). One example of subclinical atherosclerosis is carotid atherosclerosis, a condition that involves thickening of a certain layer of the wall of the carotid artery supplying the brain. This form of subclinical atherosclerosis is thought to reflect an increased risk of CHD. The use of cocaine (Lai et al. 2005) and other illicit drugs (Mondy et al. 2008) may further augment this association between HIV infection and subclinical atherosclerosis.

In addition, HIV infection may be associated with other indicators of an increased risk of CHD, including higher calcium levels in the coronary arteries (Lai et al. 2002) and dysfunction of the inner cell layer (i.e., endothelium) lining the interior surface of the blood vessels (Solages et al. 2006). Finally, HIV infection may contribute to dysfunction of the heart muscle (i.e., myocardium) (Hsue et al. 2007).

### Influence of CART on Risk

Along with HIV infection itself, its treatment using CART also appears to be a risk factor for future CHD events (Friis-Moller et al. 2003*a*). The Data Collection on Adverse Events of Anti-HIV Drugs (DAD) Study, which involved a large number of patients from several countries, demonstrated that after adjusting for traditional risk factors, CART is associated with an increased incident of MI (RR = 1.26, 95% CI = 1.12–1.41) (Friis-Moller et al. 2003*a*). Moreover, the risk of CHD appears to depend on the specific combination of medications used in CART. For example, a follow-up study to the DAD reported that one class of anti-HIV medications called protease inhibitors (PIs) was associated with an increased risk of CHD (RR = 1.16, 95% CI = 1.10–1.23), whereas another class of medications known as nonnucleo-side reverse transcriptase inhibitors (NNRTIs) was not (RR = 1.05, 95% CI = 0.98–1.13) (Friis-Moller et al. 2007). The lack of significance associated with NNRTI use, however, is difficult to interpret because most participants had to switch to different drug regimens over the course of the study, including changes in the classes of CART medications; in particular, only a few participants had relatively little exposure to PIs and therefore could serve as control subjects. Most recently, the DAD study reported that certain medications belonging to a third class of anti-HIV medications called nucleoside reverse transcriptase inhibitors (i.e., abacavir and didanosine), taken within the preceding 6 months, also were associated with MIs (RR = 1.90, 95% CI = 1.47–2.45 for abacavir and RR = 1.49, 95% CI = 1.14–1.95 for didanosine) (Sabin et al. 2008).

### Influence of HIV, CART, and Metabolic Changes on Risk

Since the introduction of CART, metabolic disturbances, such as abnormalities in the amounts of lipid in the blood (i.e., dyslipidemia) and insulin resistance, have become common among HIV-infected people. Both of these disturbances are established CVD risk factors. CART regimens, particularly those involving PIs, can alter the levels of all classes of lipids and lipid-containing proteins (i.e., lipoproteins) in the blood, especially triglyceride levels (Tsiodras et al. 2000). The exact effects on lipid profiles depend on the specific CART regimens used. For example, boosted protocols that involve two PI medications, one of which is ritonavir, can substantially increase the risk of dyslipidemia because ritonavir can contribute to dyslipidemia even at low doses (Shafran et al. 2005). It is important to note, however, that the changes in lipid levels associated with CART in some cases actually may be a “return-to-health” phenomenon rather than a harmful abnormality. In one study called the Multicenter AIDS Cohort Study (MACS) (Riddler et al. 2003), investigators found that the patients’ cholesterol and lipid values declined after the patients had been infected with HIV and before they initiated CART. During the 6 years following CART initiation, the patients’ total cholesterol levels increased slightly, but in general the levels of both “good” (HDL) and “bad” (low-density lipoprotein [LDL]) cholesterol did not exceed the levels prior to the HIV infection.

Nevertheless, the clinical consequences of the HIV- or CART-related metabolic changes can result in increased risk of CVD. For example, in the DAD study, elevated levels of total cholesterol and triglycerides, as well as presence of diabetes and abnormalities in the fat tissues (i.e., lipodystrophy), all were significantly associated with an increased risk of MI (Friis-Moller et al. 2003*a*). Moreover, CART remained a significant risk factor even after adjusting for lipid levels in the blood and diabetes; this indicates that the increased risk cannot be explained solely by CART-induced dyslipidemia or insulin resistance (Friis-Moller et al. 2003*a*).

Abnormally high cholesterol levels typically are treated using medications called statins. Statin therapy currently also is recommended to treat HIV-associated dyslipidemia, even though there are no large randomized controlled trials demonstrating that aggressive lipid-lowering therapy prevents future cardiovascular events in these patients (Oh and Hegele 2007). However, based on data in the HIV-uninfected population, statin therapy could have a profound impact on HIV dyslipidemia and future CVD events because these medications have the potential to lower lipid levels while producing anti-inflammatory effects. Only for HIV-infected patients with liver disease attributed to alcohol abuse or HCV infection, statin therapy should be considered carefully because these agents can potentially have toxic effects on the liver (i.e., hepatoxic effects).

### Influence of HIV, Inflammation, and Blood Clot Formation on Risk

As mentioned earlier, in HIV-uninfected people, biomarkers of systemic inflammation, such as C-reactive protein, assessed either individually (Danesh et al. 2005; Ridker et al. 2000) or as an aggregate multiple biomarker score (Wang et al. 2006), are related to increased cardiovascular risk. Whether these markers also are related to increased risk among HIV-infected patients still is being explored. SMART, mentioned earlier, has provided the best evidence to date that inflammation and blockage of a blood vessel resulting from a blood clot (i.e., thrombosis) are important mechanisms associated with increased CVD risk in HIV-infected people (see table 1).^6^ This analysis found that certain inflammatory markers (i.e., interleukin 6 [IL-6], d-dimer, and, to a lesser extent, C-reactive protein) were associated with an increased risk of death and a second group of markers (i.e., IL-6, amyloid P, and, to a lesser extent, d-dimer) were associated with an increased risk of CVD.

These results are consistent with two studies linking HIV to altered blood clotting (i.e., coagulation) and increased blood clot formation (i.e., thrombogenesis). In the VACS, HIV infection was associated with a 39 percent increased incidence of first venous thromboem-bolism in the pre-CART era and a 33 percent increased incidence in the CART era (Fultz et al. 2004). In another study (Schecter et al. 2001), a protein produced by the HIV (i.e., the envelope protein gp120) directly activated muscle cells in the arteries to produce a protein that initiates the cascade of reactions leading to coagulation. Together, these studies suggest that HIV may increase vascular risk triggering the inflammation and coagulation cascades.

### Influence of HCV Infection and HCV/HIV Coinfection on Risk

HCV coinfection is common among people infected with HIV (Justice et al. 2006). Whether HCV infection is an independent risk factor for CVD in this population is not clear. One study (Freiberg et al. 2007) found that among HIV-infected people with a history of AUDs the prevalence of CVD was higher among those with a HIV/HCV coinfection than among those without concurrent HCV infection. Among HIV-uninfected adults, some studies report a higher prevalence of CVD risk factors (Mehta et al. 2000), carotid atherosclerosis (Ishizaka et al. 2002), and CHD (Vassalle et al. 2004) in the presence of HCV infection, whereas other studies (Volzke et al. 2004) found no such association. These discrepancies may be explained at least in part by the absence of detailed information on alcohol consumption and other drug use and abuse.

## HIV Infection, Alcohol Use, and the Risk of CVD

Heavy drinking and AUDs are common among HIV-infected people. Based on a variety of screening tests or clinical assessments, studies have estimated that up to 40 to 50 percent of adults with HIV infection have a history of alcohol abuse or dependence (Lefevre et al. 1995; Samet et al. 2004). In a recent study involving VACS participants, 33 percent reported hazardous drinking and 21 percent had a reported diagnosis of alcohol abuse or alcohol dependence (Freiberg et al. 2010). This alcohol consumption may affect survival times, because in a computer simulation using the VACS data, moderate and hazardous drinkers had decreased survival compared with nondrinkers. Thus, for hazardous drinkers (i.e., those consuming five or more standard drinks per drinking day) with 1 or more drinking days per week overall survival was decreased by more than 3 years; with daily hazardous consumption, survival was reduced by 6.4 years (Braithwaite et al. 2007).

One mechanism through which alcohol use could influence survival in HIV-infected people is through effects on the patients’ adherence to the often complex CART and other medication regimens. One analysis of the VACS sample found that among binge drinkers, 11 percent missed medications on drinking days, 7 percent on postdrinking days, and 4 percent on nondrinking days (Braithwaite et al. 2005). Among nonbinge drinkers, these percentages were lower but still elevated compared with abstainers.

VACS investigators recently also reported that hazardous drinking and alcohol abuse and dependence were significantly associated with CVD in HIV-infected people (Freiberg et al. 2010). Among HIV-infected men, hazardous drinking (OR = 1.43, 95% CI =.05–1.94) and alcohol abuse and dependence (OR = 1.55, 95% CI = 1.07–2.23) were associated with a higher prevalence of CVD (i.e., MI, CHD, stroke, or congestive heart failure) compared with infrequent and moderate drinking. These associations persisted even after adjusting for traditional CVD risk factors, HCV infection, CART, and CD 4 count (table 2). Some association also existed with respect to specific types of CVD. Thus, hazardous drinking was also associated with a significantly higher prevalence of congestive heart failure (OR = 1.74, 95% CI = 1.04–2.91), and alcohol abuse or dependence was significantly associated with CHD (OR = 1.67, 95% CI = 1.06–2.64) and congestive heart failure (OR = 1.99, 95% CI = 1.12–3.55).

## Possible Mechanisms Underlying Alcohol’s Association With CVD Among HIV-Infected Adults

One of the consequences of HIV infection is a thinning (i.e., effacement) of the intestinal walls. Combined with the depletion of the immune cells attacked by HIV (i.e., the CD4 cells), this effacement allows bacteria living in the intestine or bacterial products (e.g., molecules called lipopolysaccharides) to leak across the gastrointestinal mucosa into the blood stream (Blagopal et al. 2008; Brenchley et al. 2006). This process is called microbial translocation. It may result in increased activation of the immune system, subsequent inflammation, and increased end-organ damage, including acute MIs and death (see the figure). Interestingly, hazardous alcohol consumption also is linked to microbial translocation among HIV-uninfected people (Keshavarzian et al. 1994; Purohit et al. 2008). Moreover, active liver disease (e.g., hepatitis resulting from alcohol use or HCV infection) may further exacerbate the effects of microbial translocation and immune activation either indirectly, because the body is no longer able to clear the microbial translocation products, or directly, because of the increased inflammation associated with the hepatitis (Balagopal et al. 2008). Similarly, it is possible that among HIV-infected and HIV/HCV-coinfected people alcohol consumption plays a major role in the progression to organ damage, CHD, and mortality.

These findings support a model proposing a two-hit hypothesis for CHD and death among HIV-infected people who consume alcohol (see the figure). In these patients, hazardous drinking combined with active chronic HIV infection or HIV/HCV coinfection leads to high levels of microbial translocation, which in turn results in increased immune activation. Increased immune activation then contributes to increased thrombosis and excessive blood clotting (i.e., hypercoagulability), which ultimately increase the risk of end-organ damage (e.g., CHD and acute MI) and death.

However, alcohol- and HIV-related microbial translocation is not the only mechanism by which the risk of cardiovascular morbidity and mortality is increased in HIV-infected, alcohol-consuming patients. As previously discussed, alcohol consumption likely influences the risk of end-organ damage and mortality by contributing to hypertension, dyslipidemia, and medication noncompliance. Finally, the risk of atherosclerosis may be accelerated particularly among heavier drinkers because of the high prevalence of cigarette smoking in this group (see the figure).

## Summary

Hazardous alcohol consumption is associated with CVD among HIV-infected people. Moreover, unlike in HIV-uninfected people, there are no data yet to suggest that moderate alcohol consumption may reduce the risk of CVD among HIV-infected people. The mechanisms by which alcohol influences cardiovascular risk among those infected with HIV are not clear; however, it is likely that both traditional risk factors (e.g., increased blood pressure and dyslipidemia) and microbial translocation play a role. With respect to the latter, alcohol may work synergistically with HIV to promote increased microbial translocation, immune activation, systemic inflammation, and thrombosis, thereby increasing the risk of future MIs. The risk of CVD may be particularly high for those HIV-infected people who consume alcohol and are coinfected with HCV.

To help reduce future cardiovascular events among HIV-infected patients, traditional risk factor modification, including lipid-lowering therapy and smoking cessation, clearly is warranted. However, because alcohol consumption may influence cardiovascular risk among HIV-infected people in multiple ways (e.g., by interacting with other established risk factors and by causing microbial translocation), successful alcohol interventions also may be important to reduce cardiovascular risk in this population.

## Figures and Tables

**Figure:**
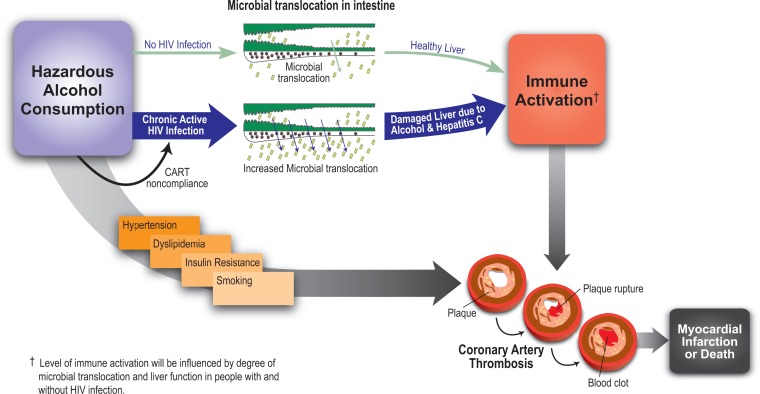
The association between alcohol consumption and cardiovascular disease. Excessive (hazardous) alcohol consumption can affect the blood vessels via its contribution to or frequent association with hypertension, abnormal levels of fat molecules (lipids) in the blood (i.e., dyslipidemia), insulin resistance, and smoking. All of these factors enhance the risk of plaque formation in the blood vessels. In addition, hazardous alcohol consumption can lead to excessive immune activation, which also can increase the risk of plaque rupture and blood clot formation, ultimately resulting in myocardial infarction and death. SOURCE: Balagopal et al. 2008; Vasan 2006.

**Table 1:** Biomarkers of Inflammation and Thrombosis and Total Mortality and Cardiovascular Disease (CVD) in SMART.

**Biomarker**	**Total Mortality**			**Fatal or Nonfatal CVD**		
	**OR**	**95% CI**	***P* value**	**OR**	**95% CI**	***P* value**
High-sensitivity C-reactive protein	3.5	1.5–8.1	0.004	1.6	0.8–3.1	0.20
Interleukin-6 (IL-6)	12.6	4.3–37.4	<0.0001	2.8	1.4–5.5	0.003
Amyloid A	2.3	0.9–5.5	0.08	1.6	0.9–2.9	0.12
Amyloid P	1.1	0.5–2.4	0.90	2.8	1.4–5.3	0.002
d-Dimer	13.3	4.4–40.3	<0.0001	2.0	1.0–3.9	0.06
Prothrombin fragment 1+2	1.4	0.6–3.5	0.45	0.8	0.4–1.6	0.56

SOURCE: Kuller et al. 2008.

NOTE: OR = odds ratio; 95% CI = 95 percent confidence interval

**Table 2:** The Association Between Alcohol Consumption and Other Covariates and Cardiovascular Disease (CVD) Among HIV-Infected Veterans.

	**Model I**	**Model II**
	Coronary heart disease (CHD) risk factor adjusted^*^ OR (95% CI)	Full model^**^ OR (95% CI)†
*n*	2422	2143‡
**Demographics**		
Age (per 10-year age-group)	1.49 (1.29–1.73)	1.53 (1.30–1.79)
Race		
White	1.0	1.0
Black	.97 (0.71–1.32)	.95 (0.67–1.34)
Hispanic	0.91 (0.54–1.53)	0.86 (0.49–1.51)
Other	1.86 (0.99–3.49)	1.80 (0.92–3.52)
More than high school education		1.53 (1.16–2.03)
**Alcohol Consumption**		
Infrequent and moderate	1.0	1.0
Hazardous	1.35 (1.01–1.79)	1.43 (1.05–1.94)
Abuse and dependence	1.51 (1.09–2.09)	1.55 (1.07–2.23)
Past drinkers (more than 12 months without a drink) vs. past drinkers (less than12 months without a drink or currently drinking)	1.31 (0.99–1.71)	1.33 (0.99–1.80)
**Cardiovascular risk factors**		
Hypercholesterolemia	2.37 (1.84–3.07)	2.36 (1.77–3.13)
Diabetes	1.58 (1.17–2.12)	1.71 (1.25–2.34)
Hypertension	3.18 (2.45–4.12)	2.94 (2.22–3.90)
Current smoking	1.80 (1.38–2.36)	1.79 (1.33–2.41)
Body mass index	0.99 (0.96–1.02)	0.99 (0.96–1.02)
**HIV-related risk factors**		
No hepatitis C and no liver disease		1.0
No hepatitis C and liver disease		1.23 (0.90–1.68)
Hepatitis C and no liver disease		1.94 (0.99–3.80)
Antiretroviral Use ‡		
Adherent		1.00
Therapy and not adherent		1.01 (0.74–1.38)
No therapy		1.05 (0.73–1.50)
**Other covariates**		
Cocaine use		1.07 (.76–1.52)
Kidney disease (glomerular filtration rate <30 ml/min/1.73m^2^)		2.39 (1.24–4.61)
Regular exercise		0.81 (0.62–1.05)

SOURCE: Freiberg et al. 2010.

NOTE:

* The coronary heart disease (CHD) risk factor model adjusts for age (in 10-year intervals), race/ethnicity, alcohol consumption, elevated cholesterol levels in the blood (i.e., hypercholesterolemia), diabetes, high blood pressure (i.e., hypertension), current smoking, and body mass index.

** The full model simultaneously adjusts for age (in 10-year intervals), race, education, alcohol consumption, hypercholesterolemia, diabetes, hypertension, current smoking, body mass index, hepatitis C and liver disease status, cocaine use, kidney disease, exercise, use of and adherence to antiretroviral therapy, and CD4 count.

† OR = odds ratio and CI = confidence interval

‡ Sample size was 2,143 for HIV-infected participants because of missing data for CD4 count and antiretroviral therapy.

## Footnotes

1 For a definition of this and other technical terms used in this article, see the glossary, pp. 288–291.

2 In patients with liver dysfunction, lactate levels in the blood increase because they are not broken down properly.

3 Apolipoprotein E is a protein involved in the breakdown of proteins with certain fat-like components (i.e., triglyceride-rich lipoproteins). Several variants of the protein (and the gene encoding it) exist; the APOE4 variant has been implicated in the development of atherosclerosis and Alzheimer’s disease.

4 Alcoholic cardiomyopathy typically is associated with a widened (i.e., dilated) left heart chamber (i.e., ventricle), with or without reduced left ventricular function.

5 CART, which also sometimes is referred to as highly active antiretroviral therapy (HAART) typically involves at least three medications from at least two types of drug classes that attack different stages of the viral life cycle and therefore are less likely to allow development of viral resistance. However, many patients over the course of their treatment develop resistance to a given drug combination and need to be switched to a different combination.

6 The study used a nested case–control design to estimate adjusted odds ratios for the fourth versus first quartile of each biomarker at baseline using logistic regression.
